# The Use of Hospital Services by Patients With Muscle Invasive Bladder Cancer in the Last Year of Life: Identifying the Areas to Improve Care

**DOI:** 10.7759/cureus.49175

**Published:** 2023-11-21

**Authors:** Monty Matson, Tony Tien, George Yardy, Paula Allchorne, James S.A. Green

**Affiliations:** 1 Department of Urology, Barts Health NHS Trust, London, GBR; 2 Department of Urology, East Suffolk and North Essex NHS Foundation Trust, Colchester, GBR

**Keywords:** mibc, quality improvement, healthcare modelling, palliative care, oncology, bladder cancer

## Abstract

Introduction: Muscle-invasive bladder cancer (MIBC) is associated with significant morbidity. However, patients’ specific health needs have not been well defined. This study analyses the utilisation of hospital resources by MIBC patients in the 12 months before death, informing healthcare modelling and enabling service redesign to improve their quality of life.

Materials and methods: All patients who died after being diagnosed with MIBC at a single hospital in the United Kingdom within four years were included. Patients’ electronic health records were reviewed to collect data on all interactions with hospital services in their last year of life.

Results: A total of 41 patients were included, with survival times ranging from one to 88 months (with a median of nine months). In the last year of life, a patient from this cohort had an average of 5.2 outpatient appointments and 2.3 emergency admissions leading to 17.1 days of inpatient stay and 1.3 operations/procedures. The most common reasons for emergency admission were for the management of haematuria (23%), urinary tract infection (23%), or chest infection (12%).

Conclusion: Patients with MIBC demonstrate significant utilisation of healthcare resources in their last year of life. An awareness of this should inform honest discussions with patients, earlier provision of palliative care, and proactive management of haematuria and urinary tract infections to improve care in this important stage of life.

## Introduction

Muscle-invasive bladder cancer (MIBC) is an aggressive malignancy with a high risk of metastasis and poor prognosis. Studies estimate a median survival time of 2.2 years, with an overall five-year survival rate of only 30% [1]. Following diagnosis, individuals often experience a significant decline in their physical, psychological, and social quality of life [2].

Amongst all malignancies, MIBC, in particular, has a large symptom burden, with haematuria, urinary frequency, and urgency causing significant morbidity [2]. This is alongside the usual symptoms of malignancy such as pain, nausea, and constipation. Studies from the United States of America (USA) have shown that this results in a high use of emergency and inpatient care in the last year of life [3]. There is a lack of evidence, however, on what health needs are driving this high utilisation of hospital care. A better understanding of the hospital services being used is the first step in improving the pathways and experience of these patients as they approach the end of life.

Specialist palliative care has been shown to significantly improve patients’ physical well-being and satisfaction as well as resulting in care being more goal concordant [4]. Despite the evidence regarding the benefits of early palliative care involvement, uptake could be optimised further with several barriers having been identified [5]. One posited barrier that is specific for MIBC is that the health needs of these patients are not well defined, despite a known high symptom burden [6].

This study aimed to assess the use of hospital services in the 12 months before death in patients with MIBC. The goal was to better inform advanced care planning, pre-empt complications of the disease, and restructure services to better manage them.

## Materials and methods

A retrospective study was undertaken using data collected from the records of all patients who were diagnosed with MIBC at a single hospital in the United Kingdom. Patients were selected from the Bladder Cancer Registry, and all patients with a diagnosis of T2-T4 bladder cancer who died over a four-year period were included. Information on the use of hospital services 12 months before death was collected from patients’ electronic health records. Data on elective admissions for planned procedures and emergency admissions for acute medical conditions were collected. Data on the reason for admission, operations, interventional radiology procedures, and radiological investigations were recorded. Outpatient appointments for urology, oncology, and other medical specialties were also collated. Palliative care referrals and reviews, including inpatient and community visits, were also included.

Causes of death were not recorded as the information from death certificates is often unreliable and difficult to validate [7]. This is less important in this study as we are seeking to identify all opportunities to improve care in patients with MIBC, regardless of whether they specifically led to death. Ethics approval was not deemed necessary due to the retrospective nature of the study and its association with a service improvement initiative. This study was completed in accordance with the Helsinki Declaration as revised in 2013 [8].

## Results

A total of 41 patients were included in this study, among which 32 (78%) had MIBC at initial diagnosis, and nine (22%) were initially diagnosed with Ta/T1 cancer that later progressed to become muscle invasive (Table 1). The median age was 79 years (range: 43-93 years) at diagnosis of MIBC and 80 years (range: 43-96 years) at death. A wide range of survival times was observed (1-88 months), with a median duration of nine months from diagnosis of MIBC to death. Three patients (7.3%) survived for two years or more after diagnosis.

**Table 1:** Demographics and bladder cancer characteristics ASA: American Society of Anaesthesiologists; WHO: World Health Organisation.

		n (%)	Median (min–max)
Age at diagnosis	<70 years	6 (14.6)	79 years (43–93)
	70-80 years	16 (39.0)	
	80-90 years	14 (34.2)	
	>90 years	5 (12.2)	
Age at death	<70 years	6 (14.6)	80 years (43–96)
	70-80 years	14 (34.2)	
	80-90 years	12 (29.3)	
	>90 years	7 (17.1)	
Time from diagnosis to death	< 3 months	9 (22.0)	9 months (1–88)
	3-6 months	4 (9.8)	
	6-12 months	11 (26.8)	
	12-24 months	14 (34.1)	
	>24 months	3 (7.3)	
ASA grade	1	1 (2.4)	2 (1–5)
	2	28 (68.3)	
	3	10 (24.4)	
	4	1 (2.4)	
	5	1 (2.4)	
WHO performance status	0	12 (29.3)	1 (0–3)
	1	12 (29.3)	
	2	14 (34.2)	
	3	3 (7.3)	

All patients’ diagnoses were confirmed with pathology specimens: 36 patients (87.8%) had urothelial cell carcinoma (UCC), and five patients (12.2%) had squamous cell carcinoma (SCC). Twelve (29.3%) patients were initially treated with curative intent, and 29 (70.7%) patients were treated with palliative intent from the point of diagnosis.

Of the 41 patients, 37 (90.2%) had at least one outpatient appointment (OPA) in the 12 months preceding death, with a total of 215 OPAs amongst the cohort. This equates to an average of 5.2 (range: 0-12) appointments per patient. Seventy-six (35.3%) appointments were with urology, 71 (33.0%) were with oncology, and 68 (31.6%) were with other specialties.

**Figure 1:**
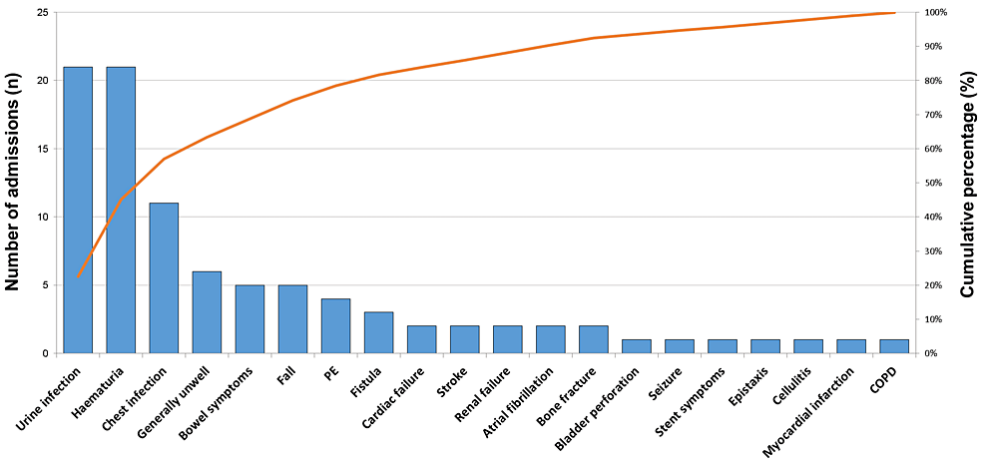
Reasons for emergency admission This figure shows a Pareto chart detailing the frequency of presentations leading to emergency admission amongst the cohort. The red line shows the cumulative frequency and demonstrates that >50% of admissions were due to urine infection, haematuria, or chest infection. PE: Pulmonary embolism; COPD: Chronic obstructive pulmonary disease.

About 33 (80.5%) patients had more than one emergency admission in the year before their death, and 16 (39.0%) had three or more. Amongst the 41 patients, there were a total of 93 emergency admissions, 31 under urology, 30 under general medicine, seven under oncology, seven under geriatrics, four under general surgery, one under intensive therapy unit (ITU), and the remaining under other specialties. These emergency admissions accounted for a total of 701 days of inpatient care, equivalent to an average of 7.5 days per admission. The frequency of different presenting complaints on admission is shown in Figure 1. The most common reasons for admission were for the management of haematuria (23% of 93 admissions), severe urinary tract infection (febrile, systemically unwell; 23% of 93 admissions), or chest infection (12% of 93 admissions).

Twenty-three (56.1%) patients were admitted electively in the 12 months preceding their deaths, five of whom were admitted on two separate occasions. Of the total 28 elective admissions, 19 (76%) were for transurethral resections of bladder tumours (TURBTs). These elective admissions accounted for a total of 82 days of inpatient care, equivalent to an average of 2.9 days per admission.

Thirty-one (75.6%) patients had at least one operation or therapeutic procedure during their last year of life. Seven (17.0%) patients had 11 emergency operations, and 14 (34.1%) patients had 23 interventional radiology (IR) procedures. Six of the emergency operations were TURBTs, two colostomies, one refashioning of a colostomy, one laparotomy and bladder repair, and one bladder washout in theatres. Of the IR procedures, 10 (24.4%) patients had nephrostomies inserted, two of whom then received a second nephrostomy sited contralaterally at a later date. Other IR procedures included three nephrostomy removals, three biopsies of lymph nodes or metastases, one radiofrequency ablation of lung metastasis, an ascitic tap, and a placement of a pelvic drain.

Electively, 17 (41.5%) patients had TURBTs (one patient had two); so, out of the 41 patients in the cohort, 20 (48.8%) had TURBTs either electively or as an emergency operation at some point in their last year of life. There were also two elective stent changes and a bladder biopsy.

Of the 12 (29.3%) patients treated with curative intent, eight (19.5%) underwent cystectomy, four (9.8%) received radiotherapy and two (4.9%) received chemotherapy. Of the 29 (70.7%) patients treated with palliative intent, 15 (36.6%) received radiotherapy, two (4.9%) received chemotherapy, and 12 (29.3%) received neither.

Thirty-three (80.5%) patients were referred to palliative care in their last year of life. On average, this referral was made two months before death (Table 2). Amongst the cohort of 41 patients, there were 65 inpatient reviews and 34 home visits by the palliative care team. Eight (19.5%) patients were admitted to a hospice and stayed for a total of 50 days. Seven of these patients died in the hospice, and one was discharged. Eight (19.5%) patients were not referred to palliative care at any point between their diagnosis and death.

**Table 2:** Summary of hospital resource use in the 12 months prior to death

Hospital resource	n (%)
*Outpatient appointments*	
Number of patients	37 (90.2% of 41 patients)
Urology appointments	76 (35.3% of 215 appointments)
Oncology appointments	71 (33.0% of 215 appointments)
Other specialties	68 (31.6% of 215 appointments)
Total appointments	215
*Emergency admissions*	
Number of patients	33 (80.5% of 41 patients)
Total admissions	93
Total inpatient stay (days)	701
*Elective admissions*	
Number of patients	23 (56.1% of 41 patients)
Total admissions	28
Total inpatient stay (days)	82
Day cases	7
*Investigations*	
Number of patients	39 (95.1% of 41 patients)
Total number of investigations	212
X-rays	92
CT scans	73
Ultrasound scans	30
MRI scans	14
Other	3
*Procedures*	
Emergency operations	11
Interventional radiology procedures	23
Elective operations	21
*Palliative care*	
Number of patients referred	33 (80.5% of 41 patients)
Time from referral to death, mean (range)	62.6 days (1-324)
Inpatient reviews	65
Home visits	34
Hospice admissions	8
Total hospice stays (days)	50

In the last year of life, a patient from this cohort had an average of 5.2 outpatient appointments (range: 0-12), 2.3 emergency admissions (range: 0-9) leading to 17.1 days of inpatient stay (range: 0-57), 0.5 elective admissions (range: 0-2), 5.2 imaging investigations (range: 0-12), and 1.3 operations/procedures (range: 0-4) per person.

## Discussion

Our data shows that patients with MIBC have considerable health needs toward the end of their lives as demonstrated by their high utilisation of hospital resources. There is limited data in the literature to compare these findings with, though a retrospective study of 6066 Medicare beneficiaries with MIBC found that 13.5% of patients had multiple admissions in their last year of life [3]. Of our cohort, 58.5% of patients had two or more emergency admissions in the same timeframe.

This large burden of emergency admissions implores us to explore to what degree these admissions are preventable. Almost half of emergency admissions were with a urological presenting complaint, principally haematuria (23%) and severe UTI (23%).

Haematuria affects nearly a third of patients with MIBC and can necessitate admission due to either anaemia or clot retention [9]. These patients are often admitted for catheterisation, bladder washout, and irrigation as well as blood transfusion, if necessary. These interventions are not always successful, however, as suggested by the wide range of admission times for patients presenting with haematuria (range: 1-33 days). Three patients (7.3%) who were admitted with haematuria on more than one occasion ultimately required transurethral resection and cystodiathermy to control the bleeding. In these cases, it may be that earlier planned intervention could have prevented the need for future admission. Several nonsurgical interventions such as hypofractionated radiotherapy, alum instillation, embolisation, and intra-arterial chemoperfusion may also have a place in reducing emergency admissions with haematuria [10]. With an ageing population that shows an increasing prevalence of atrial fibrillation and anticoagulant use [11], the need for further research in this area is pressing. With no recognised and standardised approach for managing admissions with haematuria, the British Urology Researchers in Surgical Training (BURST) research group has recently announced an international multicentre observational study to assess the impact of various practices in an attempt to establish the most effective management protocol [12].

The high incidence of severe urinary tract infections (febrile, systemically unwell) within our cohort raises the question of whether patients with MIBC are at higher risk of developing UTIs. If so, approaches such as patient-initiated oral antibiotics or antibiotic prophylaxis could be considered to reduce both morbidity and the need for admission amongst these patients [13].

Patients in our cohort had an average of 5.2 OPAs in their last year of life, 35.3% of which were with urology and 33.0% with oncology. This high number is a burden both to patients and their relatives. There is a high degree of overlap, with 28 (68.3%) patients having both a urology and oncology OPA. Multidisciplinary clinics or sequential same-day clinics may reduce this burden of healthcare on patients and improve their experience if logistical barriers can be overcome [14].

Looking at surgical operations, eight (19.5%) patients underwent cystectomies in the last 12 months of life: an operation which comes with a high degree of morbidity. These patients appear to spend more time in hospital following emergency admission than those with their bladder in situ, though our cohort was not of a sufficient size to confirm whether the difference is statistically significant. It has been shown that involvement of palliative care alongside curative therapy has the potential to reduce this morbidity, alleviate symptom burden, and reduce the need for emergency admission [15]. Despite this, none of these patients had any palliative care input alongside their surgery, and all were referred later, on an average of 75 days before death.

Of the cohort of 41 patients, 33 (80.5%) were referred to palliative care in their final year of life. This figure is significantly higher than the 4% in Hugar et al.’s US-based study, though the timing of referral was similar: two months before death in our study and between 0.9 and 2.3 months before death in Hugar and team’s study [3,6]. Given the median length of time from diagnosis to death was nine months, many of these referrals could have been made earlier. Within the cohort, eight (19.5%) patients were not referred to palliative care at any point despite an average of 257 days from diagnosis to death for this subgroup. It is unclear why referrals were not made for these patients, though several barriers are known to exist for referrals from both patients and clinicians [16]. Several barriers are less relevant to the United Kingdom than the United States, such as workforce limitations and restrictions in access to palliative care: Medicare restricts the provision of hospice and hospice-at-home care to patients with prognoses of less than six months of survival and requires patients to stop active treatments [17]. Such “mutual exclusivity” of provision does not exist in the United Kingdom, which was ranked as the top provider of palliative care internationally in the 2015 Quality of Death Index report [18]. Reasons for this ranking include the integration of palliative care into the NHS, a strong hospice movement largely funded by the charitable sector, adequate availability of specialised staff, and deep community engagement. In the United States, discussion of end-of-life care can aggravate religious sentiment that holds the sanctity of life paramount, and the report also notes that Medicare has created incentives for emergency care, inpatient stays, and intensive care, which delays the involvement of palliative care.

Whilst the guidelines of the American Society of Clinical Oncology (ASCO) recommend early palliative care for all patients with advanced malignancies, there is a lack of guidance from British or European organisations on when to involve specialist palliative care [4]. However, the English National Institute for Health and Care Excellence (NICE) recommends clinicians to engage patients with a life expectancy of less than a year in advance care planning [19]. The importance of advance care planning for these patients to ensure the care they receive is consistent with their preferences and values cannot be overstated. We hope that despite a small sample size, the results generated by this study can be used to have honest conversations with patients, their families, and carers about what to expect in their last year of life. Strengths of this paper include the breadth and detail of data extracted, allowing for in-depth analysis of hospital resource use in the 12 months before death. It utilises real-world data and pragmatically includes all patients dying with a diagnosis of MIBC to mitigate the bias or inaccuracies inherent in death certification.

A limitation of this study is its retrospective nature and the data collected from a single hospital. The data may not reflect the services in other hospitals or the care of patients in the rest of the United Kingdom. The relatively simple methodology may, however, encourage other units to assess the services for their own MIBC patients or even for other cancers to see how they can improve the care of these patients in their last year of life.

## Conclusions

With a significant symptom burden, high morbidity, and poor prognosis, patients with MIBC have considerable health needs and utilisation of hospital resources in their last year of life. Haematuria and UTI symptoms were responsible for almost half of all emergency admissions. Further research to establish the most effective management protocols for haematuria is hotly anticipated, and admissions secondary to UTIs may be preventable with patient-initiated or prophylactic antibiotics. Multidisciplinary or sequential same-day clinics can reduce the burden of OPAs on patients and their carers. Clinicians should involve specialist palliative care earlier and remain vigilant regarding patients approaching the end of life to reduce potentially unnecessary investigations and procedures. Understanding their health needs allows all clinicians to have honest conversations with these patients, and their families, about what to expect in this important last year of life.
